# Is inhibitory control a ‘no-go’ in adolescents with autism spectrum disorder?

**DOI:** 10.1186/2040-2392-5-6

**Published:** 2014-01-31

**Authors:** Anji S Vara, Elizabeth W Pang, Krissy AR Doyle-Thomas, Julie Vidal, Margot J Taylor, Evdokia Anagnostou

**Affiliations:** 1 Diagnostic Imaging and Neurology, The Hospital for Sick Children, University of Toronto, 555 University Avenue, M5G 1X8 Toronto, Ontario, Canada; 2 Neurology, The Hospital for Sick Children, University of Toronto, 555 University Avenue, M5G 1X8 Toronto, Ontario, Canada; 3 Holland Bloorview Kids Rehabilitation Hospital, University of Toronto, 150 Kilgour road, M4G1R8 Toronto, Ontario, Canada; 4 University of Toronto, 563 Spadina Crescent, Toronto M5S 2J7, Ontario, Canada; 5 Paris Descartes University and UMR CNRS 3521, Paris, France; 6 Bloorview Research Institute, University of Toronto, 150 Kilgour Road, Toronto, Ontario M4G 1R8, Canada

**Keywords:** Autism spectrum disorder, Adolescence, Brain imaging, Inhibition

## Abstract

**Background:**

Autism spectrum disorder (ASD) refers to a range of neurodevelopmental conditions characterized by social communication deficits, repetitive behaviours, and restrictive interests. Impaired inhibition has been suggested to exacerbate the core symptoms of ASD. This is particularly critical during adolescence when social skills are maturing to adult levels. Using magnetoencephalography (MEG), we identified the location and timing pattern of neural activity associated with inhibition in adolescents with autism, compared to typically developing adolescents.

**Methods:**

The MEG data from 15 adolescents with ASD and 15 age-matched controls (13 to 17 years) were collected during a go/no-go task with inverse ratios of go/no-go trials in two conditions: an inhibition condition (1:2) and a baseline condition (2:1). No-go trials from the two conditions were analyzed using beamformer source localizations from 200 ms to 400 ms post-stimulus onset. Significant activations were determined using permutation testing.

**Results:**

Adolescents with ASD recruited first the right middle frontal gyrus (200 to 250 ms) followed by the left postcentral gyrus (250 to 300 ms) and finally the left middle frontal and right medial frontal gyri (300 to 400 ms). Typically developing adolescents recruited first the left middle frontal gyrus (200 to 250 ms), followed by the left superior and inferior frontal gyri (250 to 300 ms), then the right middle temporal gyrus (300 to 350 ms), and finally the superior and precentral gyri and right inferior lobule (300 to 400 ms).

**Conclusions:**

Adolescents with ASD showed recruitment limited largely to the frontal cortex unlike typically developing adolescents who recruited parietal and temporal regions as well. These findings support the presence of an atypical, restricted inhibitory network in adolescents with ASD compared to controls.

## Background

It is theorised that impaired inhibitory control exacerbates the social deficits or repetitive behaviours and restricted interests [1-3] characterizing autism spectrum disorders (ASD) [4]. Inhibition is the ability to suppress a prepotent response and is one of many executive functions that aid in behavioural control. Relying on the prefrontal cortices of the brain, inhibition works in concert with other execution functions, such as working memory and attention, to exert top-down control on behaviour (in contrast, stimulus-driven behaviours are referred to as bottom-up processing) [5,6]. Inadequate top-down control in individuals with ASD could manifest as behaviour that is contextually inappropriate yet self-gratifying (for example, fixating on topics and objects of specific interests or repeating motor mannerisms that provide stimulation) and be indicative of impaired inhibition.

The extent of inhibitory impairment in ASD is not well understood, as the existing literature has yielded inconsistent findings. Some behavioural studies have reported inhibition deficits in ASD [1,7-11], while others have found no impairment [12-17]. This inconsistency may, in part, be due to the variety of tasks employed and the differences in task-demands [18]. These include the Stop-signal task [19], the Stroop task [15,20,21] and the anti-saccade task [9].

Neuroimaging studies have attempted to clarify the variability seen in behavioural studies by identifying possible endophenotypes within the brain. In typically developing individuals, particularly adults, the anterior cingulate cortex (ACC) and the right inferior frontal cortex have been implicated in inhibition tasks, likely related to their respective roles in error processing and top-down control. For example, Tamm *et al*. found more variability of overall brain activity in children aged 8 to 12 years compared to adults [22]. Typically developing adolescents however have been shown to activate a similar network to adults, but at a lower threshold [23].

Imaging studies on inhibition in ASD are limited, and are mainly completed in adults [24-28]. The findings from these investigations have suggested atypical overall activation patterns; however, the question remains about potential developmental differences between individuals with and without ASD. To date, only a few studies have been conducted with children [29,30] and adolescents [31]. Using a ‘Preparing to Overcome Prepotency’ task in functional Magnetic Resonance Imaging (fMRI), it was found that adolescents with ASD activated the left inferior prefrontal cortex, as well as parietal and occipital cortex significantly less than typically developing adolescents [31]. In contrast, ASD adults were reported to activate the left inferior frontal gyrus to a greater degree than control adults [24] during an fMRI go/no-go task. Also using a go/no-go task, Lee *et al*. examined the functional connectivity with fMRI of the right and left inferior frontal gyri with regions in the frontal, striatal and parietal cortices in children aged 8 to 12 years as well as adults [29]. A trend towards an interaction between age and connectivity was found between the right inferior frontal gyrus and other regions (right caudate and bilateral supplementary premotor area) in ASD [29]. In these regions, connectivity decreased with age in ASD, while in controls no change was observed with age. More recently, a magnetoencephalography (MEG) study employing a go/no-go task found decreased theta power in the anterior cingulate cortex (ACC) in children aged 7 to 14 years with ASD compared to control children [30]. Decreased ACC activity has been associated with decreased attention and poorer performance on cognitive tasks [32,33], consistent with the poorer performance seen in the ASD group of children [30].

Thus, studies have implicated the ACC and inferior frontal cortex in inhibitory control both in individuals with and without ASD. However, regions such as the parietal lobes, striatum and premotor cortex have been reported as well, but there is less of a consensus on the relevance of these regions to inhibition versus processing associated with other demands of the experimental task. As such, studies are needed to carefully characterize the inhibitory network, as well as to examine differences between adolescents with and without ASD.

Given the importance of timing in inhibition studies (for example, controlling impulsivity and anticipatory responses), neuroimaging modalities that allow measurement of the timing of brain activity add an invaluable dimension; MEG allows the determination of both the spatial and temporal patterns of brain activity [34]. Using MEG to resolve the spatiotemporal brain dynamics of inhibitory control, we adapted a go/no-go paradigm from Vidal *et al*. [35]. This paradigm had simple rules so as not to implicate working memory, and a very fast presentation rate to prevent ceiling effects, and to keep adolescents engaged and challenged during task performance. Using this go/no-go task, we have demonstrated that typically developing adolescents recruited a network that was spatiotemporally different from adults to perform the task at a comparable level (Vara *et al*., 2013; in submission).

The aims of the current study were twofold:

1. to examine differences in inhibitory control (performance measures) between adolescents with ASD and those without. We hypothesized poorer inhibition-related task performance would be seen in the ASD group compared to the control group

2. to determine the spatial and temporal brain activity associated with inhibition in adolescents with and without ASD and to compare the neural activity pattern between groups. We hypothesized that ASD adolescents would show spatially atypical and temporally delayed neural activity patterns compared to controls.

## Methods

### Participants

Included in this study were 30 adolescents. Fifteen participants (12 males) had an ASD diagnosis, between the ages of 13 and 17 years (mean age 15.5 ± 1.2 yrs; mean IQ 103.8 ± 13.6). These participants were age- and sex-matched with typically developing control adolescents (n = 15; 12 males; mean age 15.6 ± 1.3 yrs; mean IQ 112.4 ± 10.3; the control sample was included in a normative, developmental paper, Vara *et al*., in submission). None of them were on any psychotropic medications. Exclusion criteria were history of neurological disorder, including epilepsy and or acquired brain injury, known neurodevelopmental syndromes (for example, Fragile-X, tuberous sclerosis), primary psychiatric disorders (aside from ASD, for example, schizophrenia, bipolar, panic disorder), or chronic medical disorders (for example, sickle cell disease, cardiac problems, any form of cancer), current psychotropic medication use, prematurity, uncorrected vision, full scale IQ <80 and standard contraindications to MEG and MRI imaging (ferromagnetic objects in the body). All participants were recruited based on known ASD diagnosis from a clinician and the diagnosis was confirmed using the Autism Diagnostic Observational Schedule - Generic (ADOS-G) [36], and the Autism Diagnostic Interview Revised (ADI-R) [37]. Mean and standard deviation of ADOS and ADI scores for the ASD group were as follows: ADI_social_:19.4(6.4), ADI_communication_:14.8(4.8), ADI_repetitive_: 6.1(2.2); ADOS_communication_:3.1(1.4), ADOS_social_:8.4(2.1), and ADOS_repetitive_:1.45(1.43). The imaging study was approved by the institutional REB at Holland Bloorview Kids Rehabilitation Centre and the Hospital for Sick Children; the imaging was conducted at the Hospital for Sick Children. Informed consent or assent was obtained from all participants/guardians, as per institutional policies.

### Characterization measurements

*Autism Diagnostic Observation Schedule-Generic* (*ADOS-G*)

The ADOS-G [36] is a well-validated, semi-structured clinical assessment, designed to facilitate the diagnosis of ASD (Lord *et al*., [36]). It has excellent psychometric properties. Research team members with established research reliability administered either module 3 or module 4 of the ADOS-G to all of the ASD participants.

*Autism Diagnostic Interview - Revised* (*ADI-R*)

This well-established semi-structured interview [37], validated for the diagnosis of ASD, was administered by a research-reliable team member. The interview, containing 93 items, generates scores in three domains: social interaction, communication and language, and repetitive or restricted interests and behaviours.

*Wechsler Abbreviated Scales of Intelligence* (*WASI*)

Two subtests of the WASI (vocabulary and matrix reasoning) were used to obtain an abbreviated measure of IQ [38]. The two-subtest WASI is a recognized version of the WASI with comparable validity in IQ estimation to the full four-subtest WASI. The two-subtest WASI was chosen to better accommodate the participants as it allows for shorter research appointments, and therefore performance endurance was more consistent across subjects.

### Paradigm

**Figure 1:**
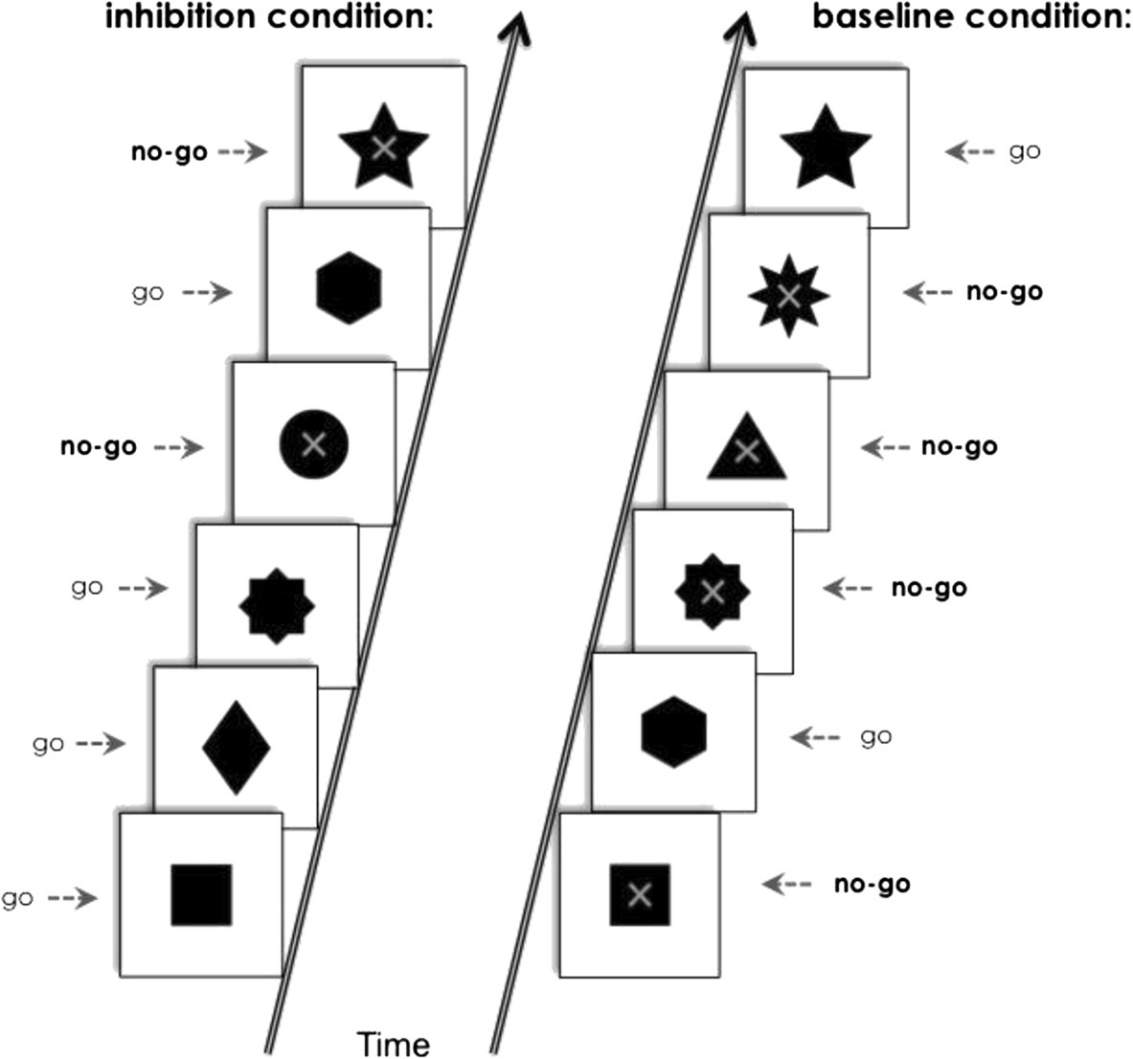
**Illustration of the go/no-go paradigm employed in this study, with the inhibition condition on the left (consisting of 33% no-go trials) and the baseline condition on the right (consisting of 67% no-go trials).** The ‘Go’ stimuli, seen as solid black shapes, and the ‘no-go’ stimuli, seen as black shapes with an X superimposed on them, are labelled.

The go/no-go task was adapted from a previous study [35] and validated in control adolescents and adults (Vara *et al*., 2013, in submission). Participants completed a ‘go/no-go’ task while lying in the MEG. Subjects were instructed to rapidly respond to go stimuli, solid black shapes on a white background and withhold their response to no-go stimuli, the same as Go stimuli, but with a grey ‘X’ superimposed in the centre (Figure 1). Two conditions were run in counterbalanced order: a baseline condition with 67% no-go trials, not encouraging a tendency to respond, and an inhibition condition with 33% no-go trials, which promoted a prepotent response tendency. To equate the behavioural performance on our task (specifically, the accuracy) across our groups, we used adaptive interstimulus intervals (ISIs) that were dependent on performance. Starting at 500 ms ISI for the first trials, the ISI was adjusted every five stimuli, where three errors or more on no-go trials would cause the ISI to increase by 100 ms, while fewer than three errors decreased the ISI by 100 ms, with the minimum ISI set to 300 ms. The stimulus duration was 200 ms.

### Behavioural measures

The mean reaction time (RT) was measured for go trials, from stimulus onset to the initial button response. RTs under 100 ms were not included, as they probably reflected subject anticipation. False alarm rate ( that is, percentage of commission errors) was calculated as the percentage of incorrect responses to no-go trials out of the total number of no-go trials. Hit rate was calculated as the percentage of correct go trials out of the total number of go trials. Statistical analyses on behavioural data were carried out using STATISTICA, Version 8, http://www.statsoft.com. A repeated measures multivariate ANOVA was run on each of the three behavioural measures - RT, false alarm rate and hit rate - to compare diagnostic group (ASD versus control) and condition type (inhibition versus baseline) effects and interactions on behavioural measures of the go/no-go task.

### Neuroimaging

MEG data were acquired on a 151-channel CTF system, using a 600 Hz sampling rate, and an online 0 to 150 Hz bandpass filter, with third order spatial gradient noise cancellation. All participants also completed an MRI scan, on a 3 T Siemens Trio system. Anatomical T1-weighted MRIs (3D MPRAGE sequence: TR/TE = 2300/2.96 ms; FA = 9°; PAT, GRAPPA = 2; FOV = 28.8x19.2 cm, 1 mm isotropic voxels) were used to co-register the MEG data. Functional analyses were conducted with scripts written in-house that generated global field power (GFPs), source localizations and permutation tests between conditions and between groups.

### Magnetoencephalography analyses

MEG trials were epoched into 600-ms windows with a 100-ms pre-stimulus baseline. Only correct no-go trials were analysed. Trials with artefacts such as eye blinks and movement were manually removed on a trial-by-trial basis, based on the agreement of two research members. The no-go trials (approximately 100 trials per participant) were averaged, and then grand averaged across participants, by condition (baseline versus inhibition) and group.

**Figure 2:**
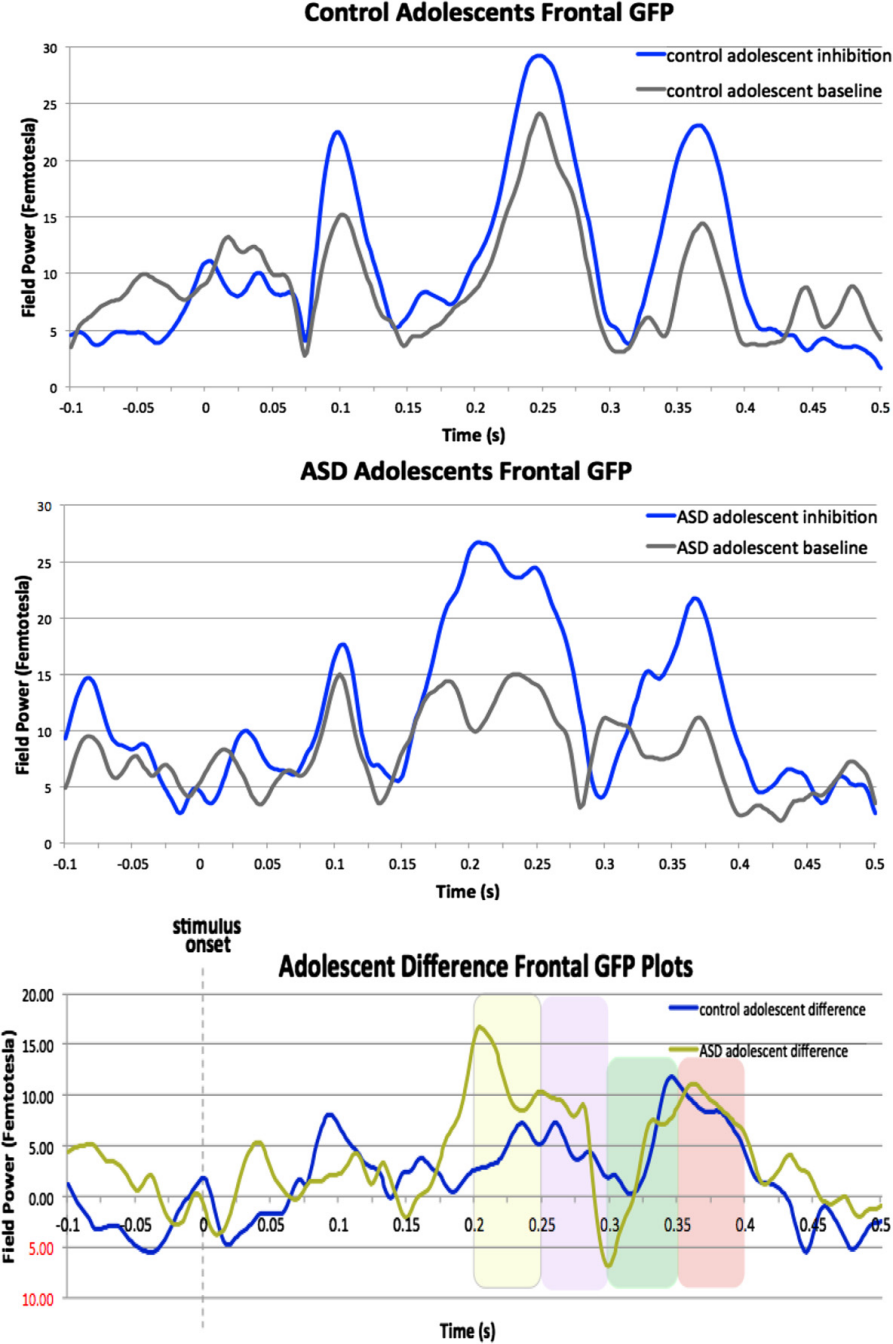
**Global field power plots from frontal sensors.** Upper plots: Global field power (GFP) plots from the frontal magnetoencephalography (MEG) sensors for the inhibition trials for the two conditions in the control adolescents (top plot) and the adolescents with autism spectrum disorder (ASD) (middle plot). Lower plot: Difference waveforms for Global field power (GFP) between the inhibition and baseline conditions, measured by MEG sensors over the frontal area of the brain on correct no-go trials for the two groups: control (blue) and ASD (gold). Stimulus onset is marked at 0 seconds. Four 50 ms time windows of interest are marked.

Global field power (GFP), which is the root mean squared power across all sensors, was calculated on our grand-averaged data, to determine MEG amplitude changes across time. To better visualize the frontal differences between groups, we generated GFP plots from the 38 frontal sensors (Figure 2). Based on the timing of the peak difference observed in these GFP plots, as well as our expectations from our normative study, in which activity related to inhibition was observed after 200 ms, we analysed the MEG data between 200 and 400 ms.

For source localisation analyses, we used an in-house vector beamformer algorithm (SPF) [39]. Multisphere headmodels were created from initial fiducial positions co-registered to each individual’s T1 MRI [40]. Data were filtered using a bandpass of 0.5 to 30 Hz and beamformer images were calculated over 50-ms non-overlapping time intervals, from 200 to 400 ms, for each condition and group. The resultant images with a spatial resolution of 5 mm were normalized to an MRI template with SPM2 (Wellcome Department of Imaging Neuroscience, London, UK). The 3D baseline condition (67% no-go) images were subtracted from those from the inhibition condition (33% no-go) to remove the visual activity [41,42].

To assess significance of within-group activity, permutation tests were completed on the subtracted beamformer images (2,048 permutations), generating activation maps at *P* < 0.005. Permutation tests shuffled group membership of the two samples, while maintaining the cardinality, and computing mean differences between the samples. The calculated mean differences for n >2,000 permutations were plotted to create a distribution, allowing the difference from the original sample to be compared to the distribution of values, to obtain *P* values. Between-group differences on the beamformer images were tested as well using permutation tests.

Images with *P* < 0.005 were visualised using MRI3DX, and only the highest 15% of peak activations were included, and with their Talairach coordinates noted; anatomical terms and Brodmann areas (BA) were verified in Talairach Client [43,44].

## Results

### Behavioural results

Mean RT, hit rate and false alarm rate are listed in Table 1 for both condition types and group types.

**Table 1:** Behavioural measures for go/no-go task for autism spectrum disorder (ASD) and control participants: mean and standard deviation (SD) for reaction time (RT), hit rate (Hit), and false alarm rate (FA)

	**Condition**	**x̅ RT (s)**	**Hit (%)**	**FA (%)**
**ASD**	**Inhibition**	361 ± 122	93.0 ± 14.3	26.5 ± 13.2
	**Baseline**	342 ± 50	88.7 ± 18.8	6.3 ± 4.1
**Controls**	**Inhibition**	309 ± 34	94.9 ± 10.8	18.1 ± 10.7
	**Baseline**	335 ± 34	96.1 ± 3.6	4.1 ± 4.8
**Effect size of group difference (eta squared)**	**Inhibition**	0.042	0.008	0.167
	**Baseline**	0.11	0.026	0.083

#### ***Reaction time***

There were no main effects of condition (inhibition or baseline conditions) (F(1,28) = 0.05, *P* = 0.825), diagnostic group (control or ASD) ([F(1,28) = 1.937, *P* = 0.175) or an interaction (F(1,28) = 2.478, *P* = 0.127) for RT.

#### ***False alarm rate***

A main effect of condition type was found (F(1,28) = 79.554, *P* = 0.001) where the inhibition condition (M = 22.3% ± 12.6 SD) evoked a higher false alarm rate than the baseline condition (M = 5.2% ± 4.6 SD). A trend towards a main effect of diagnostic group on false alarm rate was also seen (F(1,28) = 3.875, *P* = 0.059; eta squared = 0.122), where higher false alarm rates were seen in adolescents with ASD (M = 16.4% ± 8.7 SD) than controls (M = 11.1% ± 7.8 SD). The group by condition interaction was not significant (*P* = 0.110). However, because the interaction was expected to be nonsignificant for the baseline condition, we evaluated the group by condition interaction for the no-go condition. The effect of group on the false alarm rates for the no-go condition was examined and a trend towards a main effect of diagnostic group was found for the no-go condition [((1,28) = 3.737, *P* = 0.63; eta squared = .0118), where higher false alarm rates were seen in adolescents with ASD (M = 26.5% ± 3.1 SD) than in control teens (M = 18.1% ± 3.1 SD).

#### ***Hit rate***

There were no main effects of condition type (F(1,29) = 0.839, *P* = 0.367), diagnostic group (F(1,28) = 1.063, *P* = 0.311) or an interaction (F(1,29) = 2.584, *P* = 0.119) for hit rate.

### Magnetoencephalography results

#### ***Time course of global brain activity***

Waveforms representing the magnitude of overall frontal brain activity occurring from 100 ms prior to stimulus onset to 500-ms post-stimulus onset are plotted in Figure 2. These waveforms display differences of mean global field power (GFP) measured between-conditions for frontal sensors. These plots show the difference between the inhibition and baseline conditions for the control (in blue) and ASD (in gold) groups and peak differences guided our selection of time windows of interest for further analyses.

#### ***Timing and localisation of neural activity***

**Figure 3:**
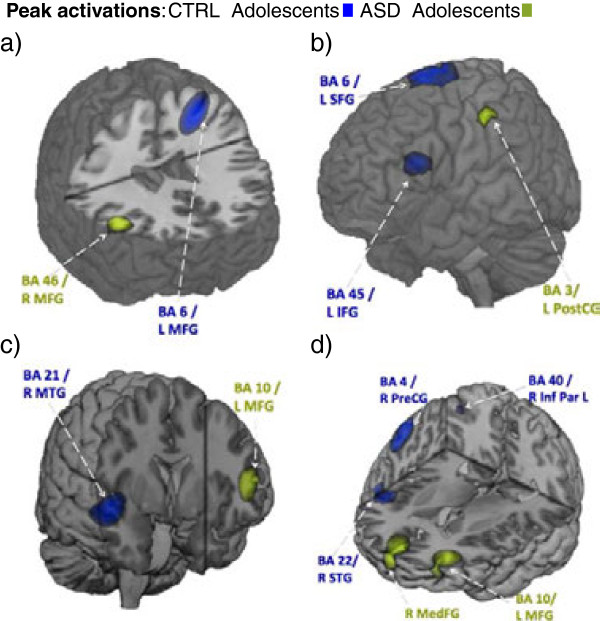
**Locations of significant (*P *< 0.005) neural activations for both autism spectrum disorder (ASD) (green) and control adolescents (blue), where the inhibition condition was greater than the baseline condition, across time windows of interest. a)** 200 to 250 ms **b)** 250 to 300 ms **c)** 300 to 350 ms **d)** 350 to 400 ms. L, left; R, right; G, gyrus; IMG, middle frontal; SFG, superior frontal; IFG, inferior frontal gyrus; MedFG, medial frontal gyrus; PostC G, postcentral gyrus; PreC G, precentral gyrus; Inf Par L, inferior parietal lobule; MTG, middle temporal gyrus; STG, superior temporal gyrus.

Significant within-group activations, where the inhibition condition was significantly greater than the baseline condition (*P* < 0.005, uncorrected; ≥5 mm from peak activation) are listed in Table 2 (with one sample, one-tailed permutation tests completed on inhibition minus baseline condition event-related beamforming (ERB) images; >2000 permutations; top 15% chosen) and displayed in Figure 3. No between-group differences were found in areas of interest (unpaired, two sample, two-tailed permutation tests, *P* < 0.005, uncorrected, 5,000 permutations).

**Table 2:** **Areas of activation (*P *** **< 0.005) during time windows of interest in control and autism spectrum disorder (ASD) adolescents**

**Time window**		**Anatomical area**	**BA**	**Talairach coordinate (x,y,z)s**		
200 to 250 ms	L	Middle frontal gyrus	6	-25	-5	55
250 to 300 ms	L	Superior frontal gyrus	6	-20	5	70
	L	Inferior frontal gyrus	45	-55	25	15
300 to 350 ms	R	Middle temporal gyrus	21	50	0	-10
350 to 400 ms	R	Precentral gyrus	4	50	-10	50
	R	Superior temporal gyrus	22	50	5	-5
	R	Inferior parietal lobule	40	40	-50	45
**Time window**		**Anatomical area**	**BA**	**Talairach coordinates (x,y,z)**		
200 to 250 ms	R	Middle frontal gyrus	46	50	25	25
250 to 300 ms	L	Postcentral gyrus	3	-55	-20	40
300 to 350 ms	L	Middle frontal gyrus	10	-30	45	5
350 to 400 ms	R	Medial frontal gyrus		5	60	0
	L	Middle frontal gyrus	10	-30	50	5

Control adolescents activated the left middle frontal gyrus (BA 6) from 200 to 250 ms followed by the left superior frontal gyrus (also BA 6) and left inferior frontal gyrus (BA 45) from 250 to 300 ms. From 300 to 350 ms, the typically developing adolescents recruited the right middle temporal gyrus (BA 21) and the right superior temporal gyrus (BA 22) from 350 to 400 ms. The right precentral gyrus (BA 4) and right inferior parietal lobule (BA 40) were also recruited during this final time window (350 to 400 ms).

The adolescents with ASD first recruited the right middle frontal gyrus (BA 45) from 200 to 250 ms followed by the left postcentral gyrus (BA 3) from 250 to 300 ms. The left middle frontal gyrus (BA 10) was then activated from 300 to 400 ms, along with the right medial frontal gyrus from 350 to 400 ms.

## Discussion

A diagnosis of ASD in our adolescent sample was associated with poorer impulsivity measures as seen in higher false alarm rates, during the inhibition condition of our task compared to the baseline condition. Neuroimaging findings complemented these behavioural findings by revealing an inhibitory network that differed significantly from the typically developing adolescent group. Adolescents with ASD recruited predominantly the frontal cortex, while controls recruited frontal as well as supplementary regions, including the inferior parietal lobule and the temporal lobe. Additionally, the ASD group initially engaged the right rather than the left frontal cortex and activated an area in the prefrontal cortex (BA 10).

### Behavioural measures across groups

No significant differences (*P* < 0.05) were found between groups for response accuracy, measured by hit rate or RT. An ASD diagnosis was associated, however, with a trend for higher false alarm rates (*P* = 0.059), or commission errors, suggesting poorer inhibitory skills in ASD individuals. A significant main effect between the baseline and inhibition conditions for false alarm rates (*P* = 0.001) confirms that the baseline condition was an effective control task, as it incurred a lower error rate.

### Magnetoencephalography results across groups

#### ***Source localization: comparing adolescents with and without ASD***

The detection of activations in this MEG study was data-driven and done on the whole-brain, thus reflecting activity in regions without a bias of prescribed ROIs. Differences between our results and those in the literature may be due in part to our analysis approach, which contrasted no-go trials in our inhibition and baseline conditions, whereas other studies contrasted no-go trials against go trials. Our method allowed for the investigation of inhibitory control without the confounding effect of the rapid motor response present in the go trials. Finally, previous studies of inhibition in ASD used fMRI, which allows little analysis of temporal processing.

#### ***Right inferior frontal activation in adolescents with ASD***

Adolescents with ASD recruited the right middle frontal gyrus in a non-homologous region (right BA 46) during the first time window of 200 to 250 ms, unlike the adolescent controls, who first engaged the left middle frontal gyrus (BA 6). The right inferior prefrontal cortex (BA 45/46), or the ventrolateral prefrontal cortex, has consistently been shown to play an important role in inhibition [45-50]. However, children and adolescents have been found to activate the right inferior frontal gyrus less than adults [51] (Vara *et al*., 2013, in submission), and typical adolescents in our current study were observed to activate the right inferior cortex at sub-threshold levels. Therefore, the significant activation of this region in adolescents with ASD, while performing more poorly on task measures of inhibition (that is, the false alarm rate) than control adolescents, suggests the activation may be inefficient or ineffective in ASD due to reduced selectivity in recruitment [52,53].

#### ***Reduced recruitment of non-frontal regions in adolescents with ASD***

Adolescents with ASD also showed activation in the postcentral gyrus (BA 3) at 250 to 300 ms, which then moved anteriorly to the left middle frontal gyrus in BA10 from 300 to 400 ms. Conversely, control adolescents recruited the inferior parietal lobe as well as the temporal lobe during final time window of 350 to 400 ms. Although the recruitment of the postcentral gyrus by adolescents with ASD was parietal, the BA 3 region is associated with somatosensory-related task activity, whereas the parietal area activated by control adolescents was the inferior parietal lobe, an area commonly activated during inhibition tasks [54-57]. It is thought that parietal activity is associated with attention, or switching attentional focus within a task [58], and greater activation in this region may reflect the better task performance [57].

The poor recruitment of more widespread cortical regions, such as the parietal [31] and temporal lobes, by adolescents with ASD may explain their difficulty in executing top-down control. The poorer performance on inhibitory tasks observed in adolescents with ASD compared to controls may result from weaker behavioural regulation of a prepotent response tendency due to atypical brain activity. As the control adolescent group recruited regions outside the frontal lobes to perform the inhibition task, it is possible that adolescents with ASD were limited to frontal lobe function due to the poorer long-range connectivity reported in ASD, which in turn hinders their ability to recruit more extensive, supplementary regions [26,52]. The greater overall frontal activity in adolescents with ASD may be related to poor long-range connectivity and local over connectivity [59], which have been proposed to underlie deficits in at least a subgroup of individuals with ASD.

#### ***BA10 activity in ASD and error monitoring***

BA 10 activity was observed between 300 to 400 ms in our adolescents with ASD, but activity was not seen in this region in the control adolescents. The BA 10 region has been associated with maintaining a balance between rapid responding and careful or controlled responding, during cognitive flexibility paradigms [60,61], as well as higher order mental representations of task contingencies [62,63]. In addition, the region within BA10 in our study corresponds to a region associated with multitasking in a meta-analysis of functional specialisation of BA 10 published by Gilbert *et al*. in 2006 [64]. We suggest that this area may have been recruited by our participants with ASD to process the probability of responding to the next trial, and the behavioural adjustment necessary to respond appropriately, or simply because of increased load. Adolescents with ASD had difficulty with the inhibition task, as measured by an increased false alarm rate, compared to control adolescents; it is possible that the BA 10 region was recruited to compensate for their poorer performance. Even with the activation of this compensatory region, individuals with ASD had more false alarms.

Limitations of this study include the small sample size, which limited our ability to detect between group differences beyond statistical doubt, as well as its cross sectional nature, which limits our ability to discuss developmental trajectories of the differences noted. In addition, comorbidity was not systematically assessed. This is important as inhibitory control defects are also a feature of ADHD (for example, [65,66]). Until recently, ADHD could not be considered as a comorbidity to ASD (based on DSM-IV criteria) and as such, these participants did not carry any such comorbid diagnoses. However, ADHD-like symptoms are common in this population and may complicate the picture. None of our participants were on stimulants or any other psychotropic medications, which suggests that they did not have significant ADHD.

## Conclusions

Inhibition-related MEG activity in the adolescents with and without ASD demonstrated distinct spatiotemporal neural processing patterns. More extensive frontal activity was found in adolescents with ASD, which we suggest may be due to inefficient long-range connectivity, [52] as well as to short-range or local over connectivity. Our argument is supported by the poor recruitment of supplementary regions in adolescents with ASD, compared to control teens who recruited parietal and temporal areas in performance of the inhibition task. Finally, BA 10 activity was found only in adolescents with ASD, between 300 and 400 ms, which could be interpreted as a compensatory response strategy in the ASD group [61-63] reflected in slower RTs yet higher false alarm rates. These findings highlight the atypical nature of the adolescent ASD inhibitory network, particularly in relation to the sequence of activations revealed using the temporal resolution of magnetoencephalography. We theorize that such atypicalities in inhibitory control may contribute to the social deficits of autism. Impulse control is necessary to function optimally within the framework of one’s social environs and impairment in social functioning during adolescence, typically a period of tremendous development in social skills, may contribute to severe social ramifications [23]. For example, the inability to suppress socially inappropriate remarks or the failure to refrain from conversations centred on one’s restricted interests, both of which are commonly observed in individuals with ASD, could lead to ostracism from peer groups. Future work needs to include large samples of longitudinal cohorts, characterized for other neurodevelopmental and neuropsychiatric comorbidities and link neurobiological findings to both associated and core symptom domains of ASD.

## Abbreviations

ACC: anterior cingulate cortex; ANOVA: analysis of variance; ASD: autism spectrum disorder; BA: Brodmann area; ERB: event-related beamforming; fMRI: functional Magnetic Resonance Imaging; GFP: global field power; ISI: interstimulus intervals; MEG: magnetoencephalography; IQ: intelligence quotient; MRI: magnetic resonance imaging; ROI: region of interest; RT: reaction time; WASI: Wechsler abbreviated scale of intelligence.

## Competing interests

EA has received consultation fees from Seaside Therapeutics and Novartis, an unrestricted grant from Sanofi-Canada, and has consulted without fees to Proximagen and Neuropharm.

## Authors’ contributions

AV participated in study coordination, subject recruitment, data acquisition, the analyses of data and the interpretation of results and drafted the manuscript. EP assisted with interpretation of results and finalizing the manuscript. KT participated in study design, participant data acquisition, and helped with drafting of the manuscript. JV contributed to study design and interpretation of results. MT contributed to the conception and design of the study and to the interpretation of results, and drafting of the manuscript. EA participated in study design and coordination, contributed to interpretation of the results and guided the drafting of the manuscript. All authors read and approved the final manuscript.
